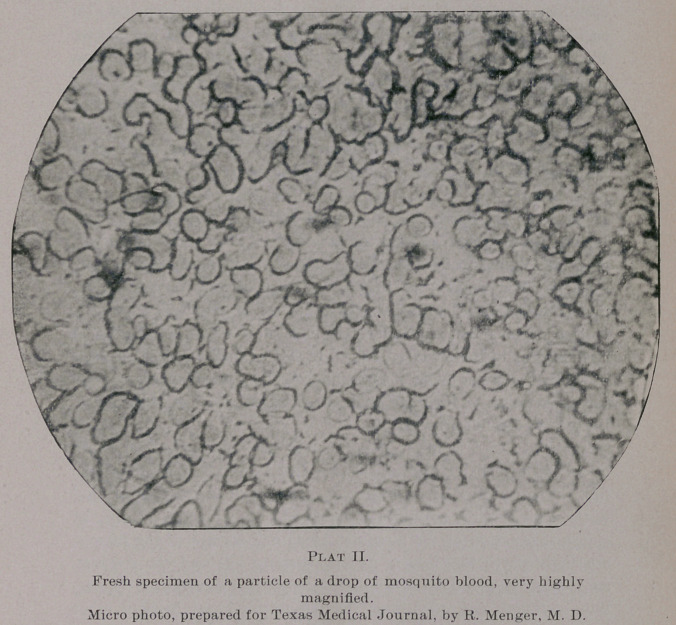# The Mosquito as a Transmitter of Micro-Organisms

**Published:** 1900-10

**Authors:** R. Menger

**Affiliations:** San Antonio, Texas


### TEXAS MEDICAL JOURNAL.

ESTABLISHED JULY, 1885.

PUBLISHED MONTHLY.—SUBSCRIPTION $1.00 A YEAR.

VOL. XVI. AUSTIN, OCTOBER, 1900. No. 4.

### Original Contributions.

For Texas Medical Journal.

### The Mosquito as a Transmitter of Micro-Organisms.

BY R. MENGER, M. D., SAN ANTONIO, TEXAS.

It is quite well known that a variety of insects, in particular *blood-sucking* insects, transmit morbid tissue-products and pathogenic bacteria upon man and animal. Some species of flies, the so-called *"blue-bottle,"* for instance, is liable to transmit anthrax bacilli, after feeding on some decaying material, upon man, and produce the malignant pustule; and the *horsefly,* with its powerful developed mouth-parts of daggers and lancets, scarifies the thick hide of animals until blood oozes from the wounds, and these scarified places often suppurate later on, and are then infested by other insects, especially the *blowfly,* which deposits numerous eggs, from which the screw-worms develop; or the small *gnatflies,* or *mosquitoes,* feed on the suppurating surfaces, transmitting pus and blood corpuscles, etc., upon other animals or man,—for instance, ophthalmitis, etc., when coming in contact with the conjunctiva of the eye. Ticks, and especially the common *"bedbugs,"* are also on record as bacteria transmitters,—notably also the tubercle bacillus from phthisical patients; and the *cattle-tick,* it is quite conclusively proven, produces the so-called Texas cattle fever through inoculation.

Now, as to the widespread *mosquito family,* scientists have accepted the theory that certain species of the genus *anopheles*

(speckled wing mosquito) are the transmitter and prime cause of malarial fevers,—spores and plasmodia being transplanted by inoculation upon human beings, and producing the malarial fever paroxysms. As yet, though, doubts are numerous as to the mosquito being the sole factor of malaria infection—climate, food and drink contamination (water and milk, etc.), or the inhalation of foul, bacteria-laden air in marshy, wet and tropical countries, etc.,—all of these influences undoubtedly bear their share in producing malaria intoxication; and still, if it be remembered how myriads of mosquitoes, of different type, abound and feed in foul water pools, sick-rooms, butcher pens, slaughter houses and hog-pens, stables, sinks, sewer canals, privy vaults and marshy regions, where

PLAT I.

A mosquito sucking-bill (a proboscis) and daggers; also a few blood corpuscles; highly magnified.

Micro photo for Texas Medical Journal, by R. Menger, M. D.

constant and lots of vegetable and bacterial decomposition is going on,—if all of these facts are taken into consideration;—also the comparative absence of the mosquito-pest and malaria-disease in salubrious and well drained and dry soils and climate; and, remembering the great inoculation facilities through the mosquito's stinging apparatus,—then, indeed, it would seem, without doubt, that the mosquito insect is a great factor and contributor to malariaintoxication. What, though, is still lacking in connection herewith, are *direct* and *positive proofs,* and, it would seem, with our modern methods of clinical and bacteriological research, it should be an easy matter, especially in tropical countries, to disclose all doubts and speculations anent this important question; but, as far as the rather meager literature at my disposal is concerned, the matter seems to be in a mere experimental state. So far, it seems, the British government has dispatched two observers, and is experiment-

PLAT II.

Fresh specimen of a particle of a drop of mosquito blood, very highly magnified.

Micro photo, prepared for Texas Medical Journal, by R. Menger, M. D.

ing in the malarious districts of the Roman Campagna with mosquito-proof huts in order to ascertain, through isolation, whether malaria is contracted through mosquito inoculation or otherwise. The result will be watched with much interest, and it is expected that by next October definite results can be obtained, and I quote the following from a late issue of the *Scientific American* on the subject:

"The physicians will not take any quinine or other precautions against the dreaded malaria. It is their intention to mix freely with the inhabitants. In Italy two million people have malaria every year, and of this number, fifteen thousand die. If the experiment proves successful, it is probable that similar houses will be built in Africa and India.

"The mosquito always exists in malarial regions, as far as has been investigated. If patients suffering from malaria come into the region, then the mosquito becomes infected and spreads the disease. Whether the insect can acquire the parasite from any other source than man has not been settled as yet. It is not probable, however; so far as it is known, malaria has never been acquired primarily in uninhabited regions. Thus explorers, after passing through a country that would naturally be supposed to be malarious, seem to be immune until they reach the coast, where the mosquitoes are abundant, and the insects are able to obtain the parasites from those suffering from the disease. An example of this is shown in Reunion Island, where there was no malaria until 1869. In that year a party of colonists came from India, and some of them suffered from malaria. The result was that the disease became very prevalent upon the island. The malaria spreader is the anopheles mosquito. It is a curious fact that they rest on a wall with their bodies at right angles to the surface, instead of flat against it, as is the case in the ordinary mosquito. The anopheles mosquito lays its eggs in stagnant water. If all the pools of stagnant water were removed, the pest would not breed."

As to our own country, it appears to me that the matter could be settled (in malarial districts) by direct and close examination: first, by examining bacteriologically samples of stagnating or foul water, on which the mosquito is supposed to feed; and, secondly, by close examination of the mosquito blood and secreta of the insects, especially particles adhering to the sucking bill and the daggers, etc. Also, it would be of great importance to ascertain if in certain instances and conditions the mosquito's blood harbors other pathogenic bacteria than the plasmodium; for instance, mosquitoes which had fed or sucked their body full with blood on persons afflicted with communicable diseases (scarlatina, yellow fever, typhoid fever, tuberculosis, etc.).

In scarlatina, where the *diplococcus,* according to W. J. Class

(*Archives of Pediatrics,* August issue, p. 624), was found in 300 cases of throat culture, and also in the blood, throat secretions, scales, etc., this micro-organism should be looked for in mosquito blood in particular. Part of the mentioned report on this subject says:

"The diplococcus scarlatina is believed to be the causative factor in scarlet fever. The germ is invariably present in the throat secretions, blood and scales of a patient having scarlatina, and it is a separate and distinct organism not heretofore described. It has been proven to be a pathogenic micro-organism, killing mice, when injected in minute quantities, in a space of time varying from less than one to twenty-four hours, according to its virulency. It produces in swine a disease whose microscopic lesions closely resemble those seen in scarlet fever, as it occurs in the human subject. The presence of blood from a patient who has just recovered from an attack of scarlet fever inhibits its growth. The subcutaneous injection of a virulent culture into guinea pigs will, under certain conditions, produce a nephritis. The blood serum of a person who has passed through scarlet fever protects an animal against the invasion of the germ. * * * In all of the 300 cases the throat culture showed the presence of the micro-organism previously described by the author. Cultures taken from the throat of a scarlet fever case will show the diplococcus scarlatina. Early in the disease it will be present in great numbers; later it will be present in diminished numbers. A number of cases are recorded to show the similarity of infection in cases of so-called "tonsilitis," scarlet fever sore throat, and scarlatina, so far as the presence of the diplococcus is concerned."

For experimental purposes, mosquitoes could easily be gathered under the mosquito bar of the respective patients, and their blood subjected to bacteriological tests. In one instance I have found bacilli resembling closely tubercle bacilli in a prepared slide of mosquito blood. The main question, though, if such tests be substantiated by others also, would be: *can a mosquito inject* (as some authors claim) *from its own body blood particles* (containing bacteria) *into another body?*—which I, as one, doubt. The mosquito is a blood "sucker," and not an "injector." Well, though, the mosquito is able to *inoculate* morbid particles adherent to the daggers and proboscis into the human capillary system during the process of stinging (*scarification*).

If a mosquito's anatomy be examined a little closer, the following presents itself: The roundish head, representing the prismatic eye-lens, is supplied at its front part with the proboscis, or sucking bill, antenæ and numerous hairy projections covered with scales. Its body shows a well developed capillary system and digestive canal, and, in the female, numerous delicate ovi-sacs containing the transparent oval eggs are seen, which, on cover-glass pressure, generally are evacuated. But the main point of interest to us is the *mouth,* or *stinging parts* of the mosquito. If a mosquito's head be mounted in glycerine and subjected to cover-glass pressure, a large amount of blood corpuscles and serum will be evacuated through the main cylindrical canal of the proboscis, and at the same time a bold, dagger-like protuberance will appear just below and in connection with the base of the mosquito's proboscis—the main "sting." Upon further pressure on the cover-glass a number of these sharp-pointed and slightly curved daggers and lancets, resembling a curved hypodermic needle, will be dislodged. (Some of these daggers and the proboscis under high-power examination, can be seen on the microphoto plate I.) These daggers seem to be under control of muscular action at the base of the proboscis, and the latter also shows, from its base up to the upper third part of the proboscis, a delicate net-work of spiral shaped contractile tissue. The end part of this proboscis (the joint part seen on the micro-photo) shows several very delicate and oval-shaped hairy pads, which, during the scarification process of the daggers, are put into oscillating motion, and serve to accumulate and absorb the extravasated blood through suction when the blood is carried through the proboscis into a system of capillary canals traversing the mosquito's body. Such, at least, is my observation in a few instances. The proboscis, as well as the daggers, is very flexible, so that the mosquito can expand or retract the same.

The accompanying micro-photo of *mosquito blood* is a rare and true reproduction of the corpuscles, magnified (under extra tube-adjustment to the photo-micrographic apparatus) over 1.200. I prepared the specimen lately from a mosquito that had its body full of blood. During cover-glass pressure a large quantity and a perfect stream of blood corpuscles were evacuated from the mosquito, besides a solid clot of blood. Quantities of blood-cells also escaped through the sucking tube and the daggers of the insect. As is to be noticed on the micro-photo, most of the cells are disintegrated, and many in a shriveled condition, and some of the latter (resembling somewhat rod bacteria, but which they are not), are undoubtedly the refractive outlines of the disintegrated cells and, others perhaps, tissue detritus. Still others show peculiar nucleation, but I am not prepared to state whether they may be considered as parasitic or not.

Further examinations may disclose more definite results, and it is to be hoped the matter may be followed up by others and arouse closer observations by those interested in microscopic research, and who have more time and better facilities to conduct such tests.

P. S.—The easiest way to gather the mosquito blood is by rupturing its body on a slide-glass or by compressing a mosquito between two cover-glasses, or by puncturing its body and pressing a small particle on the cover-glass,—dry and stain in usual manner.

## Figures and Tables

**Plat I. f1:**
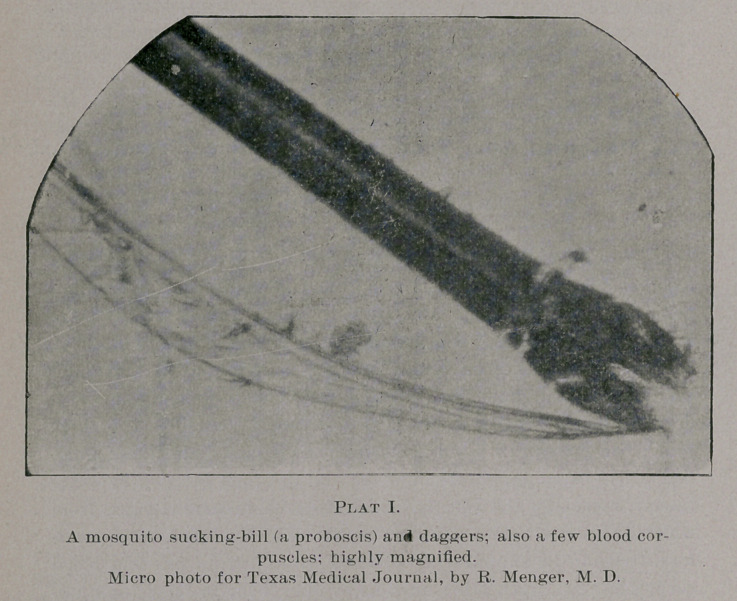


**Plat II. f2:**